# Synergistic Effect of Conditioned Medium and Calcium Phosphate Biocement on Osteogenic Properties of Composite

**DOI:** 10.3390/jfb17010010

**Published:** 2025-12-22

**Authors:** Mária Giretová, Ľubomír Medvecký, Lenka Luptáková, Radoslava Štulajterová, Tibor Sopčák, Eva Székiová

**Affiliations:** 1 Division of Functional and Hybrid Materials, Institute of Materials Research of Slovak Academy of Sciences, Watsonova 47, 040 01 Košice, Slovakia; lmedvecky@saske.sk (Ľ.M.); rstulajterova@saske.sk (R.Š.); tsopcak@saske.sk (T.S.); 2 Department of Biology and Physiology, University of Veterinary Medicine and Pharmacy in Košice, Komenského 73, 041 81 Košice, Slovakia; lenka.luptakova@uvlf.sk; 3 Institute of Neurobiology of Biomedical Research Center, Slovak Academy of Sciences, Šoltesovej 4–6, 040 01 Košice, Slovakia; szekiova@saske.sk

**Keywords:** rat mesenchymal stem cells, biocomposites, calcium sulfate hemihydrate, osteogenesis

## Abstract

The aim of the study was to investigate the synergistic effect of conditioned medium (CM) and two types of calcium phosphate biocements on the osteogenic properties of a composite material through rat bone marrow-derived mesenchymal stem cells (MSCs). Briefly, MSCs were cultured for 7 and 17 days in extracts derived from the two biocement types. These extracts were supplemented with 5% (*v*/*v*) of concentrated CM. The CM was obtained from rat bone marrow MSC cultures after a 48 h conditioning period. The results showed that the addition of CM had a significant positive impact on the osteoblastic differentiation of MSCs, particularly in the extracts from the tetracalcium phosphate/monetite/calcium sulfate hemihydrate biocement (designated as CAS cement) compared to the other tested cement extract (designated C cement). After 17 days of culturing, a notable increase in cell viability and alkaline phosphatase (ALP) activity, as well as the upregulation of osteoblastic-related gene expression, was found. This enhancement in osteogenic activity was likely driven by the growth factors and bioactive molecules present in the CM. The study concluded that supplementing the biocement extracts with only 5% of 10X concentrated CM is sufficient to significantly influence and improve the in vitro characteristics, cell behavior, gene expression, and synthesis of cell products. It was demonstrated that, especially in the CAS supplemented with CM (CAS + CM) extract system, the improvement in osteogenic properties was due to the synergistic effect between the higher concentration of calcium ions in extracts released from the calcium sulfate hemihydrate-containing cement and the bioactive molecules supplied by the CM.

## 1. Introduction

Biocements based on calcium phosphates belong to an important field of biomaterials intended for reconstruction and regeneration of hard tissues (bone, cartilage) in veterinary and human medicine for decades. Recognized for their excellent biocompatibility and capacity to promote bone growth (osteoconductivity), these cements characterize the self-setting process in which a mixture of a basic compound (such as tetracalcium phosphate (TTCP)) and an acidic compound (like brushite or monetite) reacts using liquid component calcium-deficient hydroxyapatite (CDHA) [1]. The appeal of calcium phosphate cements (CPCs) lies in their inherent bioactivity, non-toxic nature, and, critically, their bioresorbable quality, allowing for the complete replacement of the implant with natural, new bone over time. A wide range of molecules can be incorporated into biocements to refine and manage their characteristics. These additives encompass both naturally derived substances (chitin, chitosan, alginate, gelatin, amino acids, cellulose, collagen) and synthetic polymers, specifically poly (lactic-co-glycolic acid), polycaprolactone, poly (L-lactic acid), and polyethylene glycol [2,3].

Addressing bone defects resulting from trauma (fractures), pathology (tumors, infections), or developmental abnormalities (congenital diseases) remains a significant clinical challenge across both orthopedic and oral and maxillofacial surgery. Large-scale bone lesions are unable to repair spontaneously, necessitating external intervention [4].

Calcium sulfate hemihydrate (CSH) is an inexpensive and plentiful raw material. While its main drawbacks as a bone filler include poor handling, weak mechanical properties, and a rapid resorption rate for some uses, it is advantageous because it can modulate osteoblast-related gene expression and serve as a delivery vehicle for growth factors and antibiotics [5]. The incorporation of CSH into hydroxyapatite (HAP) cement demonstrated significant enhancement in in vivo properties. Following implantation in rat muscle, the material exhibited good biocompatibility and underwent gradual bioresorption over a 12-week period, accompanied by blood vessel penetration. While the calcium sulfate phase was resorbed after 6 weeks, no evidence of bone formation was observed at either the 6-week or 12-week mark [6].

Tissue engineering has emerged as a promising strategy for the repair and reconstruction of bone injuries. This field utilizes a combination of diverse biomaterials, the development of three-dimensional porous matrices and scaffolds, and the application of osteoprogenitor cells and growth factors to stimulate and facilitate natural tissue regeneration [7]. Mesenchymal stem cells (MSCs) can be isolated from postnatal organs of animals, including humans. In the body, they represent a reservoir of readily available reparative cells capable of mobilization, proliferation, and differentiation into the required cell type in response to certain signals from damaged tissues (inflammation, necrosis, etc.) [8]. They have excellent proliferative capacity while maintaining their multidifferentiation capacity [9]. Under appropriate conditions, they are capable of differentiating into cells of various phenotypes. They exhibit immunomodulatory and anti-inflammatory therapeutic effects and paracrine activity by secreting biologically active molecules [10].

Mesenchymal stem cells MSCs have demonstrated vast potential across various regenerative medicine applications. Their therapeutic power is not just about the cells themselves but mainly stems from the rich and complex blend of factors they secrete, which actively promote tissue repair. A new, compelling approach is using MSCs’ conditioned medium (CM), a culture medium without the addition of animal serum, in which stem cells proliferate and secrete biologically active substances through various pathways, which simply harvests these beneficial secretions. MSC-CM is rapidly becoming a preferred, safer alternative to implanting the cells directly while maintaining strong therapeutic efficacy [11,12].

The current literature demonstrates that using the stem cell-conditioned medium, which includes the full array of bioactive factors secreted by the cultured cells, provides a regenerative effect equivalent to that of cell transplantation [13]. MSC-CM, which delivers beneficial bioactive molecules, has demonstrated its therapeutic potential across a broad spectrum of medical conditions. These applications include ischemic diseases (like stroke and myocardial infarction), neurodegenerative disorders, type 2 diabetes mellitus, spinal cord injuries, alopecia, acute and chronic wounds, male infertility, and injuries to the liver, lungs, periodontal tissues, and musculoskeletal system [14,15,16].

The use of CM has several advantages over the use of stem cells, as CM can be easily obtained, stored at −80 °C, lyophilized, packaged, and transported. Since there are no cells in it, the relationship between the MSC donor and the final recipient of CM does not have to be autologous [17]. Beta-tricalcium (β-TCP) phosphate mixed with CM obtained from the culture of human bone marrow-derived MSCs was implanted into atrophic sites of the maxilla after tooth extraction for the purpose of bone augmentation for the insertion of a dental implant, and after 6 months of healing, significantly higher vascularization and the formation of new bone tissue in the jaw was confirmed compared to the applied cement without CM [16]. Similarly, a positive effect of exosomes from CM from human MSC culture on osteoconductivity and bone regeneration during the healing of a created defect in the skull of rats was found after mixing with β-TCP and implantation in vivo. It is assumed that exosomes increase the osteoinductive ability of β-TCP, which causes subsequent activation of endogenous MSCs at the defect site in animals and significantly stimulates defect healing. In vitro tests have shown that exosomes can be released from β-TCP and can be taken up by human MSCs during culture and significantly enhance the proliferation, migration, and osteogenic differentiation of MSCs [18]. Quade et al. (2020) introduced a novel concept for bone defect healing utilizing biomimetic mineralized collagen scaffolds. These scaffolds were functionalized with a central depot containing a signaling factor-enriched cocktail derived from hypoxia-conditioned human mesenchymal stem cells (hMSCs). The study confirmed the scaffold’s angiogenic potential—as determined by co-culturing human umbilical vein endothelial cells (HUVECs) with osteogenically induced human bone marrow stromal cells (hBMSCs)—by observing prevascular structures sprouting throughout the interconnected pores of the collagen matrix [19]. CM from bone marrow stem cells immobilized on the surface of titanium dental implants, which were subsequently transferred to rat femurs, showing excellent results of implant integration and stabilization [20].

Atelocollagen scaffold with CM and exosomes significantly influenced the formation of new bone tissue during the healing of artificially created defects in the rat skull, and the results indicated the connection between angiogenesis and osteogenesis [21]. Sun et al. (2012) examined the impact of prepared CM from rat MSC culture on primary rat osteoblasts in in vitro culture with different ratios of CM and culture medium with fetal bovine serum (FBS) (proliferation, gene expression, Western blot, ALP activity) [22].

This secretome in CM includes growth factors, soluble proteins, lipids, mRNA, microRNA, cytokines, chemokines, and even components of the extracellular matrix. Specifically, stem cell-conditioned medium (CM) is a rich source of crucial growth factors and cytokines. These include vascular endothelial growth factor (VEGF), Platelet-derived Growth Factor (PDGF), Hepatocyte Growth Factor (HGF), basic Fibroblast Growth Factor (bFGF), Macrophage-Stimulating Protein (MSP), Keratinocyte Growth Factor (KGF), and Insulin-like Growth Factor 1 (IGF-1). These components are active participants in cell regeneration and angiogenesis (new blood vessel formation) [23,24,25]. Transforming Growth Factor Beta (TGF β) plays a critical and complex role in osteogenesis (bone formation) and bone remodeling. It is one of the most abundant growth factors stored in the bone matrix [26]. VEGF is recognized as one of the most critical mediators for pro-angiogenesis. Its significance in the wound healing process extends beyond this, as it also drives fibroblast proliferation and the associated collagen synthesis, which are essential for tissue repair [27].

The objective of our study was to investigate the biological and physicochemical properties of biocement extracts, supplemented with a relatively easily obtained conditioned medium (CM) derived from rat mesenchymal stem cell cultures. The synergistic effect of bioactive factors in CM and calcium ions released from two types of CPC cements (TTCP/monetite based biocement (C) and C cement mixture with calcium sulfate hemihydrate (CSH) additive (CAS)) on the expression of osteogenic differentiation-related genes in cells in vitro and other osteogenic related characteristics (ALP activity, collagen production, viability) may provide novel perspectives in regenerative bone defect therapy.

The primary objective was to investigate the possible in vitro synergistic activity resulting from the combination of extracts of two different calcium phosphate cements (C) and (CAS) with relatively easily obtained conditioned medium (CM) derived from rat mesenchymal stem cells (MSCs) containing bioactive molecules and soluble factors. Specifically, we focused on how the mutual interaction between biofactors in CM and ions released from biocements affect osteogenic differentiation (gene expression, ALP activity, collagen production, and viability) of MSC cultures in vitro. This work offers a new possible perspective on the development of CPC-based materials in combination with bioactive molecules contained in CM, potentially opening new perspectives in the regenerative therapy of bone defects.

## 2. Materials and Methods

### 2.1. Biocement Preparation

The C cement and CAS were prepared according to refs. [1] and [28], respectively. Briefly, the tetracalcium phosphate/monetite/calcium sulfate hemihydrate (TTCPM/CSH) powder cement mixture was prepared by the in situ reaction between TTCP and a diluted solution of the orthophosphoric acid (86% analytical grade, Merck, Darmstadt, Germany)/H_2_SO_4_ (96%, analytical grade, Merck) mixture in 80 *v*/*v* % ethanol (reaction solution) using a planetary ball mill with agate balls and a vessel for 30 min at room temperature. In the case of C cement, no H_2_SO_4_ was added to the reaction mixture. The final Ca/P mole ratio in the C cement was close to 1.67, which corresponds to the Ca/P mole ratio in hydroxyapatite. The CAS contained 5 wt % of CSH. The cement pastes were prepared after mixing a TTCPM powder mixture with 2% solution of NaH_2_PO_4_ (as a liquid component) at a P/L ratio = 2. The molded samples (6 mm *D* × 12 mm *H*, pellet form) were prepared for mechanical testing. The release test was performed on samples prepared by molding cement pastes into a stainless steel cylindrical mold (6 mm *D* × 12 mm *H*), and the samples (350 mg cement/10 mL solution) were soaked in 0.9% NaCl solution with pH = 7.4/37 °C for selected soaking times of 4, 24, 72, and 168 h. The concentration of released ions in the solution was determined by ICP OES (Horiba Activa, HORIBA Jobin Yvon Inc., Park Avenue, Edison, NJ, USA) after filtration through a membrane filter (PVDF, pore size 45 µm, Millipore, Tulagreen, Ireland).

The microstructures of cement surfaces were observed by a field emission scanning electron microscopy (JEOL FE SEM JSM-7000F, Tokyo, Japan) equipped with an EDX analyzer (Oxford instruments, Abingdon, UK) after coating with carbon. The phase compositions of the samples were analyzed by Fourier transform infrared spectroscopy (FTIR) (Shimadzu, Kyoto, Japan, IRAffinity1, 400 mg KBr + 1 mg sample).

### 2.2. Cell Culture, Cytotoxicity, Live/Dead Staining, Calcium Deposits, and Collagen I Staining

Rat MSCs from rat femur and tibia bone marrow were used and characterized for in vitro experiments [29]. Cadavers of Wistar rats (300 g) used as controls for other experimental purposes, for MSC isolation, were provided by the Institute of Neurobiology of BRC of SAS.

MSCs from passage 2 were plated in wells of a 48-well plate (Brand, Wertheim, Germany) at a density of 2 × 10^4^ cells/400 µL of culture medium/well. The cells were maintained in an incubator for a period of 24 h at 37 °C, 95% humidity, and 5% CO_2_ until they reached a semi-confluent monolayer stage. After 24 h of culturing, the culture medium was discarded and replaced by appropriate extracts. All experiments were conducted in triplicate. The cells cultured in the extract-free complete culture medium served as the negative control (NK).

Calcium phosphate cements (C and CAS) were immersed in a complete cell culture medium. This medium consisted of α-modification Eagle’s minimum essential medium (EMEM; Biosera, Marikina, Philippines) supplemented with 10% fetal bovine serum (FBS), 1% antibiotic-antimycotic solution, and an osteogenic cocktail. The osteogenic supplements included L-ascorbic acid (50 µg/mL), dexamethasone (50 nM), and β-glycerophosphate (10 mM) (all from Sigma-Aldrich, Saint Louis, MO, USA) at a ratio of 0.2 g/mL for 24 h at 37 °C [30,31]. For long-term cytotoxicity evaluation and determination of osteogenic differentiation of the cells (up to 17 days of cultivation), the cements were diluted with culture medium at a ratio of 1:1. The conditioned medium (CM) labeled P2 48 h 10X conc. was added at a concentration of 5% (*v*/*v*) of the total extract volume ((labeled C + CM) and (CAS + CM)) and was refreshed twice a week concurrently with a complete extract change. The methodology for obtaining and characterizing CM is described in Section 2.3.

The cytotoxicity was determined after 7 and 17 days of cultivation of rat MSCs in extracts supplemented with CM (labeled C + CM and CAS + CM) and CM-free extracts (labeled C and CAS) by the MTS test (Cell titer aqueous one solution cell proliferation assay, Promega, Madison, WI, USA). The absorbance of the final products metabolized by enzymatically active live cells was measured by a UV VIS spectrophotometer at 490 nm (Shimadzu, Kyoto, Japan). The proliferation of cells was expressed as a percentage of measured absorbance of the experimental groups (treated cells) in relation to the measured absorbance of negative control cells (NK cells cultivated in culture medium supplemented with osteogenic supplements, extract-free, CM-free). The quantity, morphology, and density of cells growing in the mentioned extracts were visualized by live/dead cell staining. Fluorescein diacetate stains live cells green, and propidium iodide is capable of passing through the compromised membranes of non-viable cells, resulting in red staining of these cells. The images were captured by a fluorescence microscope (Leica DM IL LED, blue filter, Heerbrugg, Switzerland).

Collagen produced by osteoblastically differentiated cells was stained using the Picrosirius Red method, and the presence of hydroxyapatite deposits was visualized by Alizarin Red staining. These stainings were performed after 17 days of rat MSC cultivation in biocement extracts with and without conditioned medium (CM) addition. Once the staining solution was removed and the cells were washed, they were subsequently visualized using light microscopy (Leica DM IL LED, Heerbrugg, Switzerland).

### 2.3. Conditioned Medium Characterization

Conditioned medium (CM) was obtained from a culture of rat MSCs cultured in 75 cm^2^ culture flasks (amount 10 mL/flask) at passage 2 in FBS-free, phenol red-free medium Dulbecco’s Modified Eagle’s Medium low glucose (DMEM LG, Sigma Aldrich, St. Louis, MO, USA) for 48 h (labeled as: P2 48 h 10X conc.). The obtained medium was centrifuged (400 g/10 min) and filtered through 0.22 µm filters (Merck Millipore, Tullagreen, IRL) and concentrated 10X using Amicon 3 kDa (Merck, Darmstadt, Germany) filter units according to the manufacturer’s instructions. The protein content was determined by Bradford assay. The CM was added at a concentration of 5% (*v*/*v*) of the total extract volume (C + CM) and (CAS + CM) and was refreshed twice a week concurrently with a complete extract change (cytotoxicity, ALP activity, gene expression, staining).

Quantitative detection of rat VEGF and rat TGFβ in conditioned media (CMs) was performed using an ELISA assay (Invitrogen, Carlsbad, CA, USA). The assay was conducted on the conditioned medium utilized in the present experiment (CM P2, 48 h conditioning, concentrated 10X) and also on CMs obtained from different passages of rat MSC cultivation (P3, P4, P5) as well as various conditioning time lengths (24 h, 48 h, and 72 h). These latter CMs were not concentrated.

Our goal was to monitor the influence of the passage number and conditioning time length on the quantitative indicators of the given factors secreted by MSCs.

The ELISA assay was performed according to the manufacturer’s recommendations. Samples were run in duplicate for each condition, and the assay was repeated three times.

### 2.4. Gene Expression

The assessment of gene expression followed a methodology analogous to the one detailed in Reference [3]. Briefly, for the extraction of total RNA, we initiated the process with approximately 10^6^ cells per sample. Total RNA was isolated from the cultured cells using the RNeasy Mini Kit (Qiagen, Germantown, MD, USA), strictly adhering to the manufacturer’s protocol. Residual genomic DNA (gDNA) was eliminated by digestion with an RNase-free DNase set (Qiagen, Germantown, MD, USA). The purity and concentration (yield) of the extracted RNA were subsequently determined using a NanoDrop spectrophotometer (Thermo Scientific, Madison, WI, USA). The synthesis of complementary DNA (cDNA) was conducted using the RT2 First Strand Kit (Qiagen, Germantown, MD, USA). The synthesized cDNA was then carried forward for quantitative real-time PCR (qPCR) experiments. The quantification of target genes in the cDNA samples employed specific primers for rat genes (detailed in Table 1). Each qPCR reaction mixture contained triplicate cDNA samples, the specific primer mix for the gene of interest, and the RT2 SYBR Green qPCR master mix (Qiagen, Germantown, MD, USA). Reactions were prepared in a 96-well plate (Roche, Basel, Switzerland), sealed with an optical adhesive cover (Roche, Switzerland), and run on a real-time PCR system machine (Roche, Switzerland).

The thermocycling profile was as follows. Initial denaturation/incubation lasted for 10 min at 95 °C, and amplification (45 cycles) was performed at 95 °C for 15 s, followed by 60 °C for 1 min. Specificity of the amplification was verified post-run by generating a melting curve.

Rat β-actin cDNA served as the endogenous housekeeping control. Relative gene expression (fold change) in biomaterial-treated cells compared to untreated control cells was calculated using the comparative method, specifically the formula 2^−ΔΔCT^ method.

### 2.5. ALP Activity

The ALP activity of osteoblasts differentiated from rat MSC cultivated in the above-mentioned extracts (Section 2.2) with CM addition and CM-free extracts was determined in cell lysates, which were prepared by freezing (−20 °C) and subsequent centrifugation of the thawed samples. The assay involved incubating the cell supernatant with p-nitrophenyl phosphate in diethanolamine buffer (pH 9.8) at 37 °C for 60 min. The resulting p-nitrophenol (produced during the ALP enzyme catalysis) was quantified spectrophotometrically at 405 nm against a calibration curve. The ALP activities were normalized to protein content, expressed as µmol of p-nitrophenol produced per min per µg of protein. Protein quantification utilized the Bradford method with Coomassie blue G250 [37]. The results (*n* = 4) were subjected to one-way ANOVA analysis for statistical evaluation, at level α = 0.05.

## 3. Results

### 3.1. Cement Properties

**Figure 1:**
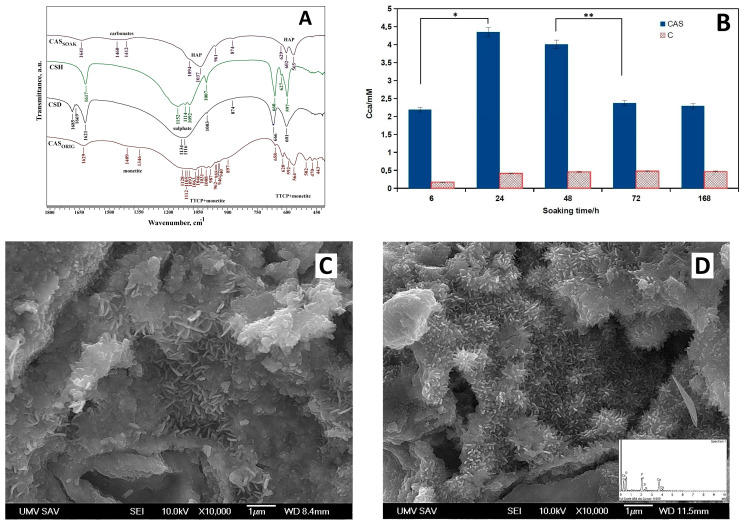
(**A**) FTIR spectra of the CAS powder (CAS_soak_-soaked in SBF solution, CAS_orig_-original CAS powder); (**B**) release of calcium ions during soaking in saline (* *p* < 4 × 10^−5^; ** *p* < 0.005); (**C**) SEM micrograph of C cement; (**D**) SEM micrograph of CAS cement.

FTIR spectra analysis of the original CAS powder (Figure 1A) identified stretching and bending vibrations of the phosphate group in TTCP (1063, 1031, 1008, 987 cm^−1^, and 962–941 cm^−1^), as well as bending vibrations of the P–O and P–O(H) monetite bonds (1409 and 1346 cm^−1^) and bending vibrations of SO_4_(ν_1_), SO_4_(ν_4_), and H_2_O (658 and 1629 cm^−1^) in CSH [38,39,40]. After soaking the cements in SBF solution, carbonate hydroxyapatite was found in both cements, with traces of CSD in the CAS cement, which is consistent with the EDX analysis of the CAS cement, where the composition contained a small amount of sulfur.

The other peaks show very low intensity, which means that they are poorly distinguishable due to the low sulfate content. At the same time, following 7 days of immersion in simulated body fluid (SBF), TTCP and monetite transformed into nanohydroxyapatite with a weakly recognizable peak from the OH stretching vibrations. The release of calcium ions from both cements during soaking in saline is shown in Figure 1B. The comparison clearly showed a rapid increase in calcium concentration in CAS solution due to the dissolution of CSH following the reduction during the transformation from CSH to gypsum (calcium sulphate dihydrate—CSD), which has lower solubility than the original CSH powder. The calcium concentration was more than ten times lower in the C solution because calcium ions formed by TTCP hydrolysis interact with phosphate ions arising from monetite to almost insoluble calcium-deficient hydroxyapatite as the final product of the cement transformation. It should be noted that the maximum pH after 196 h of soaking was 7.7 for CAS cements and 8.0 for C cements.

Fine needle-like hydroxyapatite particles were found in the microstructures of both cements (Figure 1C,D), and a more detailed analysis clearly showed that particle refinement could be seen when CSH (or CSD after hydration) was present in C cement. This fact was probably a result of the much higher calcium concentration and deeper supersaturation of the surrounding environment around the cement particles relative to hydroxyapatite and the formation of a larger number of nucleation sites for hydroxyapatite precipitation.

### 3.2. Proliferation, Live/Dead Staining, ALP Activity, Calcium Deposits, and Collagen I Staining

**Figure 2:**
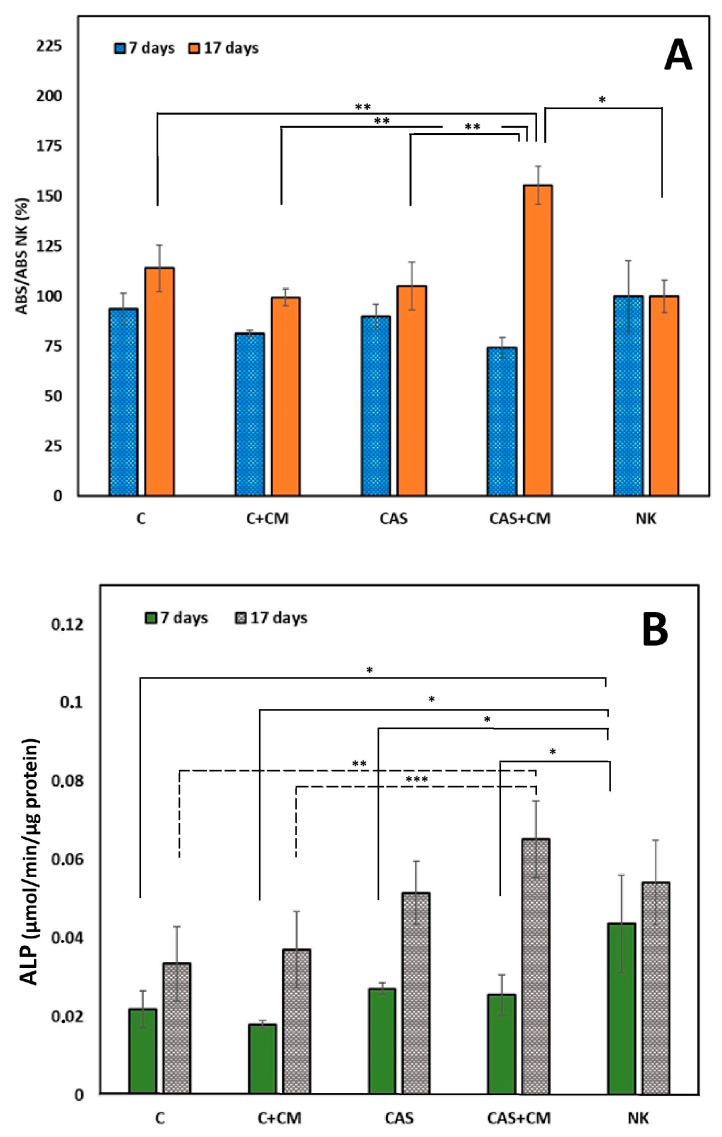
(**A**) Proliferation of MSCs in cement extracts after 7 and 17 days of cultivation (* *p* < 0.007; ** *p* < 0.01); (**B**) ALP activity of MSCs in cement extracts after 7 and 17 days of culturing (* *p* < 0.05; ** *p* < 0.009; *** *p* < 0.013).

As shown in Figure 2A, after 7 days of cultivation, reduced cell proliferation compared to NK is noticeable in all tested cultures. During prolonged cultivation, an increase in cell proliferation in all tested extracts was found. Higher proliferation compared to NK was only revealed in CAS + CM extracts (statistically significant difference, *p* < 0.007). The statistically significant difference (*p* < 0.01) between C, C + CM, and CAS compared to CAS + CM after 17 days of cultivation indicates a synergistic effect as a result of enhanced calcium ion concentration and the bioactive factors originating from CM acting on cells cultured in CAS + CM extracts.

In the case of the ALP activity of differentiated cells in all cement extracts (Figure 2B), a decrease in the means of ALP activity was indicated after 7 days of cultivation compared to NK (statistically significant difference, *p* < 0.05). After 17 days of cultivation, despite a rise in ALP activity, no statistically significant difference in mean ALP activities was found between C and C + CM or CAS groups, as well as CAS + CM and NK groups, contrary to statistically significant differences between C (*p* < 0.009) or C + CM (*p* < 0.013) and CAS + CM.

**Figure 3:**
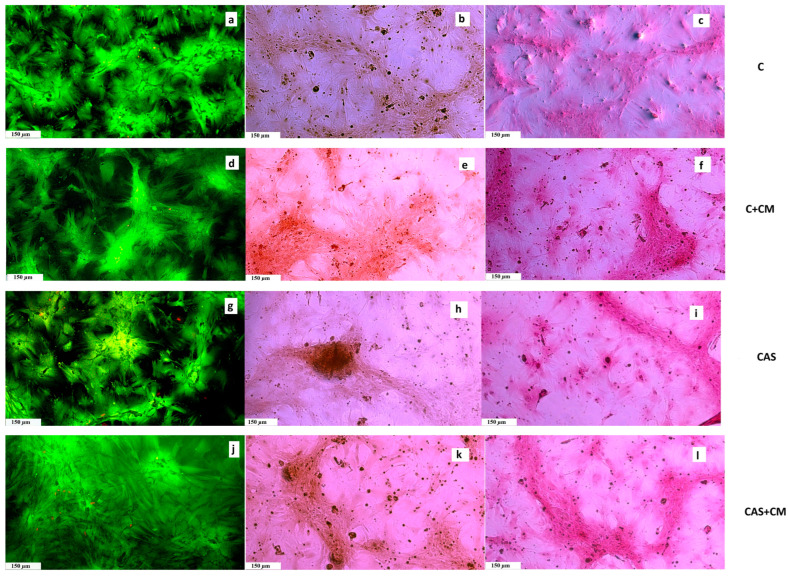
(**a**,**d**,**g**,**j**) Live/dead staining of cells growing in cement extracts (live cells - green, dead cells - red) after 17 days of cultivation; (**b**,**e**,**h**,**k**) Alizarin Red staining of calcium deposits produced by cells in extracts after 17 days of cultivation; (**c**,**f**,**i**,**l**) Picrosirius Red staining of collagen produced by cells cultivated in extracts after 17 days of cultivation.

Throughout all experimental groups, we observed very similar findings characteristic of non-cytotoxic cement extracts, where well-spread and interconnected cells formed a dense monolayer with increasing population density upon prolonged cultivation (Figure 3). The captured images are in agreement with the data from the MTS proliferation assay.

In all tested samples, we noticed and captured collagen produced by osteoblasts as part of the extracellular matrix (ECM) after 17 days of cultivation. Furthermore, the production of calcium deposits was clearly identified in the wells by Alizarin Red staining.

#### 3.2.1. Conditioned Medium Characterization

**Figure 4:**
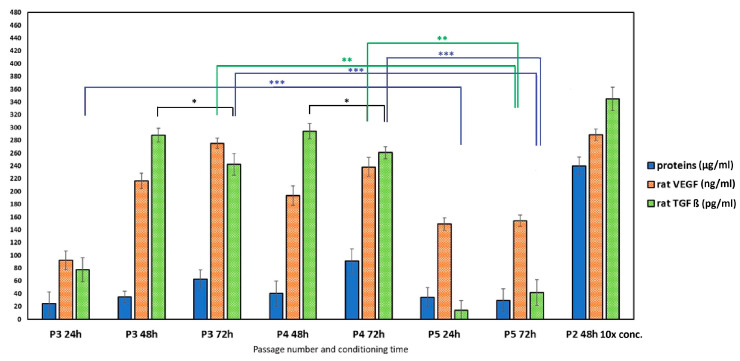
The concentration of proteins, rat VEGF, and rat TGFβ in conditioned media obtained from rat MSC cultivation (* *p* < 0.05; ** *p* < 0.001; *** *p* < 0.0001).

The content of proteins in conditioned media (Figure 4) increased with culture time in P3 and P4 passages; the same finding can be applied to the concentration of VEGF, contrary to TGFβ, with a slight decrease after 72 h of culturing (statistically significant difference between 48 and 72 h groups, *p* < 0.05). The passage 5 rat MSCs produce a statistically significantly lower amount of VEGF (*p* < 0.001) and TGFβ (*p* < 0.0001) in relation to P3 and P4 cells. This is probably due to the higher cell passage of P5 cells, which is associated with the onset of senescence and possible reduction in growth factor secretion.

#### 3.2.2. Gene Expression

**Figure 5:**
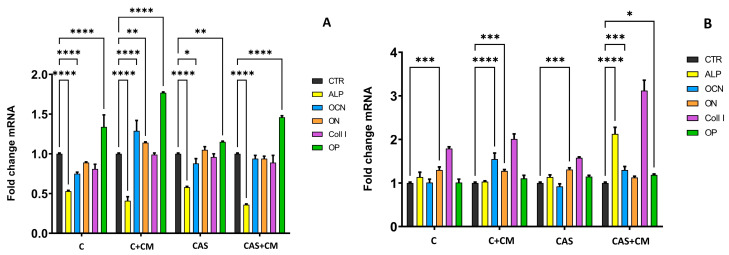
Relative gene expression after 7 days (**A**) and 17 days (**B**) of culturing rat BMMSC in extracts (* *p* ≤ 0.05; ** *p* ≤ 0.01; *** *p* ≤ 0.001; **** *p* ≤ 0.0001); CTR-negative control sample.

For the evaluation of the osteoblastic activity of differentiated MSCs, growing in extracts free of CM and extracts with CM addition, the osteogenic gene expressions for ALP, OCN, OP, ON, and COLL I were evaluated using RT-qPCR analysis compared to the control sample (CTR cells growing in culture medium supplemented with osteogenic supplements, extract-free, CM-free). The relative gene expression examined after 7 days of culturing (Figure 5A) identified a statistically significant downregulation of ALP genes in all tested samples. A statistically significant upregulation of OCN, ON, and OP genes was observed in the C + CM sample. In the CAS and CAS + CM samples, a statistically significant upregulation of only OP gene expression was determined. OCN, ON, and COLL1 gene expressions in the case of cells growing in CAS + CM extracts were statistically non-significant.

On the other hand, after 17 days of culturing (Figure 5B), a significant upregulation of OCN, ON, and COLL1 was observed in cells growing in C + CM extracts, and a significant upregulation of ALP, OCN, OP, and COLL1 genes was determined in cells growing in CAS extract with CM addition. ALP gene expression was, after 17 days of culturing, statistically non-significant in C, C + CM, and CAS extracts cultured cells. Nevertheless, the ON gene was upregulated in all samples after 17 days, except for the CAS + CM sample. OP and ALP gene expressions were non-significant in the C + CM sample. OCN gene expression was upregulated in C and CAS cement with CM addition, but in C and CAS cement extract cells, the expression of the OCN gene was non-significant.

## 4. Discussion

Currently, there are very few studies focused on analyzing the influence of CM on the in vitro properties of calcium phosphate-based biocements and on the behavior of cells grown in the CM-enriched biocement environment. Richter et al. (2023) studied a CPC scaffold combined with mesoporous bioactive glass functionalized with hypoxia CM (HCM). HCM-functionalized scaffolds in relation to non-HCM scaffolds showed an improved cell proliferation in vitro and the highest rate of new bone formation in vivo [41]. Sun et al. (2012) showed that MSC-CM also remarkably decreased OCN gene expression, especially in the case when the cells were cultured in MSC-CM compared to pure DMEM. On the other hand, the proliferation of the cells was not significantly different between the MSC-CM groups and the control group. The expression of OCN is an important indicator of the osteoblastic differentiation of cells, as well as increased levels of OPN, which is another marker indicating osteoblast differentiation [22].

Maxson et al. (2010) reported that conditioned medium (CM) acts as a modulator of fate. Specifically, CM from differentiating chondrocytes promoted osteogenic differentiation, and CM from differentiating osteoblasts promoted chondrogenic differentiation. The researchers concluded that these influences are probably due to secreted soluble factors [42]. Zhong et al. (2019) found that mouse osteoblast-conditioned medium combined with basic medium at a ratio of 3:7 administered to induced pluripotent stem cells (iPSC-MSCs) resulted in an enhancement of osteogenic differentiation, a rise in alkaline phosphatase activity, and osteogenic marker Runx2 expression [43]. Yun et al. (2024) developed a three-dimensional fabricated Ca-Si scaffold immersed in polycaprolactone (PCL) coated with exosomes. The scaffold was transplanted into a mouse calvarial bone defect [44]. Zhang et al. (2016) found that the exosome/β-TCP combination scaffolds possess better osteogenesis activity than pure β-TCP scaffolds [18].

Although many scientific teams are focusing on exosomes in regenerative medicine, on the other hand, however, using CM directly has some advantages over using exosomes. The biologically active molecules secreted by stem cells are a complex mix. They contain the entire secretome, which includes not only exosomes but also various soluble factors essential for regeneration. CM is much simpler and cheaper to produce than isolated exosomes. It only requires cell culture and subsequent filtration/concentration. Exosome isolation requires specialized, expensive, and time-consuming techniques, like ultracentrifugation or specialized kits. Both CM and exosomes represent promising cell-free therapies that avoid the risks (like immune rejection or microvascular occlusion) associated with transplanting the whole stem cell [21,45,46].

The overexpression of COLL 1, ALP, and ON genes was found in MSCs after long-term cultivation of up to 14 days in TTCP/monetite/calcium sulfate hemihydrate cement extracts. The comparison between gene expressions in cells cultured in extracts from pure calcium phosphate (C cement) and composite cement composed of calcium phosphate cement and calcium sulfate hemihydrate addition revealed significantly higher gene expressions of ALP, osteonectin (ON), and osteopontin (OP) in cells cultured in composite than in C cement extracts [28]. Alkaline phosphatase (ALP) is significantly expressed in the cells of mineralized tissue and performs a critical function in hard tissue formation. It directly facilitates mineralization by increasing the local concentrations of inorganic phosphate. ALP enzyme activity typically increases significantly and reaches its maximum relatively early in the osteogenic differentiation process (often around days 7–14 in in vitro culture). As the MSCs progress to the final stage of maturation and matrix mineralization (late stage), ALP activity generally begins to decline, while later markers, like Osteocalcin (OCN) and calcium deposition, increase [47]. This finding supports our results, showing the upregulation of the OCN gene expression in cells cultured in C and CAS extracts supplemented with CM after 17 days of culturing. The ALP gene expression was upregulated in CAS + CM extract-growing cells. It was found that higher concentrations of Ca^2+^ ions demonstrated a concentration-dependent effect on osteoblasts. Proliferation was enhanced at 2–6 mM, and differentiation and matrix mineralization improved with concentrations up to 10 mM [48]. Osteonectin (ON, also known as Secreted Protein Acidic and Rich in Cysteine or SPARC) exhibits a high affinity for type I collagen, the primary protein component of the bone matrix. By binding to collagen, it helps to organize the collagen scaffold, preparing it for the subsequent deposition of the calcium phosphate component [49].

A significant upregulation of ON gene expression was observed in our tested samples after 17 days of culturing. Osteopontin (OP) is primarily a secreted, highly phosphorylated glycoprotein, rich in Arginine–Glycine–Aspartate. The function of osteopontin in MSC osteogenic differentiation is multifunctional, involving cell matrix adhesion, lineage determination, and crystal formation [50]. After 7 days of culturing, a statistically significant upregulation of OP gene expression was found in all tested samples, regardless of CM addition. After 17 days of cultivation, the upregulation of COLL1 and ALP genes was identified in C + CM and CAS + CM samples. Collagen type I is the most abundant protein in the body and a major structural component of the extracellular matrix (ECM). Its gene expression is a key indicator of fibroblast and osteoblast activity. Is considered a marker of osteogenic differentiation in MSCs, differentiating into osteoblasts [51]. Cells growing in cement extracts supplemented with CM clearly showed higher ability for osteogenic differentiation in relation to CM-free extracts.

Transforming growth factor-β (TGFβ) signaling is considered to be active in pro-osteogenic early stages of MSC differentiation, often by promoting MSC proliferation to the osteoprogenitor lineage. In the later stages, during osteoblast maturation, mineralization, and transition into osteocytes, TGFβ signaling can become an inhibitor. This late-stage inhibition is thought to be a mechanism to prevent over-mineralization [52]. TGFβ is recognized as one of the most abundant cytokines within the bone matrix. Its family members, bone morphogenetic proteins (BMPs), are known for their ability to induce bone formation both in vitro and in vivo. Crucially, signaling cross-talk between the BMP and TGFβ pathways plays a vital role in regulating osteoblastic differentiation. Consequently, TGFβ inhibitors may prove invaluable for treating various bone diseases by accelerating BMP-induced osteogenesis [53]. Vascular endothelial growth factor (VEGF) has a coupled role in the osteogenic differentiation of MSCs. Its primary function is to promote angiogenesis (blood vessel formation), which is absolutely essential for the subsequent formation and maintenance of new bones [54]. VEGF has been shown to promote the proliferation and migration of MSCs, which is important for concentrating the progenitor cells at a site of bone repair [55].

Figure 4 illustrates the concentration of TGFβ and VEGF within the examined conditioned media. The sets of examined conditioned media differed from each other by the mesenchymal stem cell passage number and the duration of the conditioning time (24 h, 48 h, 72 h). The results demonstrated that the concentration of both VEGF and TGFβ increased with longer conditioning times. Furthermore, a statistically significant decrease in the amount of both growth factors was observed in the passage 5 (P5) CM compared to the concentrations determined in the P2, P3, and P4 CM. This finding is consistent with the phenomenon of replicative senescence (aging in culture) often exhibited by MSCs at higher passage numbers, which is frequently associated with a decline in the secretion of key paracrine factors, including VEGF and TGFβ [56].

It was found that the addition of conditioned medium had a strong effect on osteoblastically differentiated rat MSCs in extracts from CAS biocement in relation to other tested cement extracts. An increase in ALP activity and an upregulation of osteoblastic-related gene expression, as well as an increase in cell viability, were observed after 17 days of culturing. The increase is probably mediated by growth factors and bioactive molecules obtained in conditioned medium. We determined that only a 5 (*v*/*v*) % addition of 10X concentrated CM into biocement extracts significantly influences and improves the in vitro osteogenic characteristics, including the cell behavior, gene expression, and composition of the extracellular matrix, especially in CAS + CM cement extracts. It is probably caused by the synergistic effect of calcium ions released from cement with the addition of CSH and bioactive molecules from CM on cultured rat MSC cells.

## 5. Conclusions

This research demonstrates a significant synergistic enhancement of osteogenic capabilities when a concentrated conditioned medium is combined with extracts from (CAS) biocement in an in vitro model using rat mesenchymal stem cells (MSCs). The key finding is that the addition of only 5% (*v*/*v*) of 10X concentrated CM to the CAS extracts markedly improves osteogenic outcomes after 17 days. This includes a notable increase in alkaline phosphatase activity, the upregulation of osteoblastic gene expression, enhanced cell viability, and improved synthesis of extracellular matrix components. This effect is attributed to a synergy between the calcium ions released from the CAS cement and the growth factors/bioactive molecules present in the CM. It could be hypothesized that by utilizing the synergistic effect, this material has the potential to accelerate the bone healing process by quickly promoting osteoblast differentiation and ECM synthesis at the defect site, potentially reducing patient recovery time. The practical suitability of this composite biocement for achieving enhanced bone regeneration in critical-size defects must be verified in an in vivo environment, which will form the primary focus of our further research.

## Figures and Tables

**Table 1:** Forward and reverse primers of genes used for RT-PCR experiments.

Genes	Primers (5′3′)	Reference
B-actin rat	F: GTAGCCATCCAGGCTGTGTT R: CCCTCATAGATGGGCAGAGT	[32]
Type I collagen rat (COLL1)	F: CCAGCTGACCTTCCTGCGCC R: CGGTGTGACTCGTGCAGCCA	[33]
Osteocalcin rat (OCN)	F: ACAGACAAGTCCCACACAGCAACT R: CCTGCTTGGACATGAAGGCTTTGT	[34]
Osteopontin rat (OP)	F: CCGATGAATCTGATGAGTCCTT R: TCCAGCTGACTTGACTCATGG	[35]
Osteonectin rat (ON)	F: GGAAGCTGCAGAAGAGATGG R: TGCACACCTTTTCAAACTCG	[35]
Alkaline phosphatase rat (ALP)	F: AACCTGACTGACCCTTCCCTCT R: TCAATCCTGCCTCCTTCCACTA	[36]

## Data Availability

The original contributions presented in the study are included in the article, further inquiries can be directed to the corresponding author.
